# A Case Report of Crigler-Najjar Syndrome Type I and Schizophrenia: Exploring the Intersection of Rare Metabolic and Psychiatric Disorders

**DOI:** 10.7759/cureus.98936

**Published:** 2025-12-10

**Authors:** Haya Manasrah, Abdulrahman H AlQaderi, Mehnaz Z Ali

**Affiliations:** 1 Medicine and Surgery, Emirates Health Services (EHS), Dubai, ARE; 2 Psychiatry, Emirates Health Services (EHS), Dubai, ARE; 3 Psychiatry, Al Amal Psychiatric Hospital, Dubai, ARE

**Keywords:** crigler-najjar syndrome type i, kernicterus, liver transplantation, metabolic disorders, neuropsychiatric disorders, neurotoxicity, oxidative stress, schizophrenia, ugt1a1 mutation, unconjugated hyperbilirubinemia

## Abstract

Crigler-Najjar syndrome type I (CN-I) is a rare autosomal recessive disorder caused by mutations in the UGT1A1 gene, leading to a deficiency of bilirubin-UDP-glucuronosyltransferase and resulting in severe unconjugated hyperbilirubinemia.

Unconjugated bilirubin accumulation can cause neurotoxic effects and has been implicated in oxidative stress within the brain, potentially influencing neuropsychiatric disorders such as schizophrenia.

We present the case of a 38-year-old male diagnosed with CN-I at birth after presenting with neonatal jaundice and subsequent kernicterus. Despite normal early development, he experienced progressive deterioration in speech, gait, and cognitive abilities.

At age 13, he underwent a partial liver transplant. Several years post-transplant, he developed schizophrenia, exhibiting hallucinations, persecutory delusions, irritability, and behavioral disturbances, resulting in multiple psychiatric admissions and ongoing treatment with paliperidone and olanzapine. Family history revealed a brother with Crigler-Najjar syndrome type II and parental consanguinity.

This case suggests that bilirubin dysregulation in CN-I may contribute to schizophrenia via oxidative stress and neurotoxicity in brain regions critical for cognitive and motor functions, and that post-liver transplantation metabolic changes may further influence psychopathology. It underscores the need to consider metabolic disorders in the pathogenesis of psychiatric illness and supports a potential role for bilirubin in schizophrenia.

Further research is warranted to explore the pathophysiological connections between metabolic disorders and neuropsychiatric manifestations.

## Introduction

Crigler-Najjar syndrome type I (CN-I) is a rare autosomal recessive genetic disorder resulting from mutations in the UGT1A1 gene. This gene encodes the enzyme bilirubin-UDP-glucuronosyltransferase, which is responsible for the conjugation of bilirubin with glucuronic acid, a crucial step in bilirubin detoxification. Mutations in UGT1A1 also contribute to related disorders, such as Crigler-Najjar syndrome type II and Gilbert syndrome. CN-I is characterized by severe hyperbilirubinemia, as the enzyme deficiency leads to an accumulation of unconjugated bilirubin in the bloodstream due to impaired conjugation. This buildup of unconjugated bilirubin manifests clinically as persistent jaundice, a hallmark feature of the condition. Unlike type I, Crigler-Najjar syndrome type II involves partial enzyme activity and presents with milder hyperbilirubinemia that typically responds to phenobarbital [1].

The incidence of CN-I is estimated to range between one in 750,000 and one in 1,000,000 individuals [2,3]. Notably, unconjugated bilirubin acts as an antioxidant, and its role in oxidative stress within the brain has been hypothesized to influence neuropsychiatric disorders such as schizophrenia. Schizophrenia is a chronic, severe mental disorder characterized by disturbances in perception, thought, emotion, behaviour, and cognition, typically presenting with positive symptoms such as delusions and hallucinations, negative symptoms such as social withdrawal and diminished emotional expression, and marked cognitive deficits. It usually begins in late adolescence or early adulthood and has a multifactorial etiology, with a strong genetic component and consistent evidence of structural and functional brain abnormalities on neuroimaging [2]. Research has demonstrated variability in bilirubin levels among patients with schizophrenia, with elevated levels being observed during acute psychotic episodes [2]. This variability suggests a potential correlation between bilirubin fluctuations and the severity of schizophrenia symptoms, indicating that bilirubin may play a more dynamic role in the pathophysiology of schizophrenia serving beyond a mere biomarker [3].

This report presents a case of Crigler-Najjar syndrome type I in a patient with coexisting schizophrenia, with a focus on exploring the intersection of these two conditions. The discussion aims to provide a comprehensive analysis of the potential interactions between Crigler-Najjar syndrome type I and schizophrenia, highlighting the complex pathophysiological interplay and the associated challenges in clinical management. Through this case, we seek to offer deeper insights into the nuanced relationship between metabolic disorders and neuropsychiatric conditions.

## Case presentation

A 38-year-old male with a lifelong diagnosis of CN-I, confirmed through gene sequencing, presented in the neonatal period with severe unconjugated hyperbilirubinemia and jaundice. Within the first weeks of life, he developed clinical features consistent with acute bilirubin encephalopathy, including lethargy, poor feeding, abnormal posturing, and axial hypotonia, and was diagnosed with kernicterus on the basis of these neurological signs with total serum bilirubin of 480 µmol/L (28 mg/dL), predominantly unconjugated.

Early motor and language milestones were reportedly age-appropriate, with no developmental delays documented in childhood; he attended mainstream schooling and was independent in basic activities of daily living.

At age 13 (in 1998), he underwent a partial liver transplantation for CN-I. Following transplantation, his jaundice resolved, and serial liver function tests, including total bilirubin (5.3-6.6 µmol/L), have remained within normal limits, indicating good graft function and sustained correction of his hyperbilirubinemia.

Approximately six years later, in late adolescence (age 19), his family noted a gradual deterioration in functioning, with changes in his voice and speech clarity, a less steady gait, and a decline in attention, memory, and problem-solving abilities. This deterioration was accompanied by increasing irritability and behavioural dysregulation, including episodes of shouting, verbal aggression and occasional violent outbursts towards family members, neglect of self-care and poor hygiene during acute phases, reduced sleep and appetite, and recurrent crises triggered by frustration over sexual dysfunction. His psychotic symptoms were characterised by persecutory delusions, such as repeatedly expressing that “some people want to harm him,” and auditory hallucinations, inferred from frequent episodes of talking and laughing to himself in the absence of external stimuli. These symptoms ultimately led to a diagnosis of schizophrenia and a marked loss of academic, occupational, and social functioning.

In terms of psychosocial history, he became engaged in early adulthood and later married; he currently lives in the community with his wife, with whom he has one child. Marital and sexual difficulties, particularly erectile dysfunction, have been prominent stressors and frequently precipitate irritability and behavioural crises, alongside occasional jealousy and suspicious interpretations of others’ behaviour towards his wife. Educationally, he attended mainstream primary school up to approximately Grade 6 and did not complete secondary or higher education or any formal vocational training. Occupationally, he previously worked as a farmer but has been unemployed for several years in the context of chronic schizophrenia, cognitive-behavioural deterioration, and the need for continuous community psychiatric and home-care support. Socially, he lives with his wife but remains heavily dependent on his mother and extended family for daily support, medication supervision, and crisis activation; he is independent in basic activities of daily living yet markedly impaired in social and occupational functioning, and he denies the use of tobacco, alcohol, or illicit substances.

He has required multiple hospital admissions due to recurrent episodes of hallucinations and paranoia and is managed by the community home care team for continuation of treatment and regular monitoring of adverse effects and physical health. Currently, his condition is well managed with paliperidone palmitate long-acting injection 150 mg intramuscularly every four weeks (following an earlier regimen of 350 mg every three months) and oral olanzapine 10 mg at night, with short-term as-needed increases up to 10-20 mg/day during acute exacerbations of irritability and aggression. In addition to pharmacological treatment, he has been followed by an intensive community psychiatry and home-care service, receiving regular home visits, crisis intervention, and ongoing monitoring of adherence, risk, and family dynamics. His management has included psychoeducation about schizophrenia and his comorbid medical conditions, as well as family-focused sessions involving his mother and wife to address behavioural crises, medication supervision, and the impact of his symptoms on the household. An interdisciplinary plan of care has been implemented, with input from psychiatry, nursing, primary care, and urology, including specialist assessment and management of his sexual and urinary symptoms. At the time of the current evaluation, routine laboratory investigations, including liver function tests, were within normal limits, physical and basic neurological examinations were unremarkable, and no brain imaging or formal psychometric/neuropsychological testing was performed.

The clinical evaluation and longitudinal psychiatric follow-up were conducted in the Community Psychiatry Department, Behavioral Health Services, within a home-care/community psychiatry programme. His care has involved a multidisciplinary team including adult psychiatrists, community psychiatric nurses, crisis and home-treatment staff, emergency department physicians, family medicine/primary care physicians, and a consultant urologist managing his sexual and urinary complaints.

Family history was notable for a brother diagnosed with Crigler-Najjar syndrome type II who underwent liver transplantation in early childhood, before any clinical evidence of kernicterus or bilirubin-induced neurological complications. In contrast to our patient, his brother subsequently achieved normal motor and cognitive milestones and progressed through mainstream schooling with good academic performance. The patient’s parents are first cousins, indicating a consanguineous background and increased genetic risk for autosomal recessive disorders.

This case presents a unique opportunity to explore the potential association between metabolic disorders such as Crigler-Najjar syndrome, bilirubin dysregulation, and the exacerbation of schizophrenia and psychiatric manifestations, highlighting the need for further investigation into the correlation of bilirubin dysregulation and psychiatric prognosis of individuals with metabolic syndromes.

## Discussion

The intersection of CN-I and schizophrenia presents an opportunity to explore the complex relationships between metabolic dysregulation, neurotoxicity, and psychiatric disorders. This case highlights the potential role of bilirubin in the pathogenesis of schizophrenia and underscores the broader implications of metabolic disorders in psychiatric health.

Role of bilirubin in neurodevelopment and cognitive function

Bilirubin, a byproduct of heme metabolism, is typically conjugated by the enzyme UGT1A1 for safe excretion. However, in conditions like CN-I, where bilirubin conjugation is impaired, unconjugated bilirubin accumulates in the bloodstream, crossing the blood-brain barrier and exerting neurotoxic effects [1]. Kernicterus, a hallmark of severe hyperbilirubinemia, causes irreversible damage to brain regions such as the basal ganglia, hippocampus, and cerebellum - areas critical for motor, cognitive, and emotional regulation [4]. This damage may predispose individuals to neurocognitive deficits, including impaired executive function and impulse control, which are common in schizophrenia. Furthermore, bilirubin-induced neurotoxicity during neonatal periods can have lasting impacts on brain plasticity and connectivity, potentially setting the stage for psychiatric vulnerabilities later in life [4].

Oxidative stress and neuropsychiatric disorders

A central theme in the pathogenesis of schizophrenia is oxidative stress, a mechanism strongly implicated in this case. While bilirubin possesses antioxidant properties at physiological levels, elevated unconjugated bilirubin acts paradoxically as a neurotoxin by promoting oxidative damage. This is particularly significant in the context of CN-I, where chronic hyperbilirubinemia can exacerbate oxidative stress and neuroinflammation, disrupting critical neurotransmitter systems, including dopaminergic and glutamatergic pathways [5]. These disruptions contribute to the cognitive dysfunction and psychosis observed in schizophrenia. Moreover, studies have demonstrated significantly higher bilirubin levels during acute psychotic episodes, suggesting a dynamic interplay where bilirubin fluctuations might influence symptom severity [2]. This evidence positions bilirubin not only as a biomarker of psychiatric states but also as a potential driver of disease mechanisms.

Genetic insights and familial patterns

The genetic underpinnings of CN-I, involving mutations in the UGT1A1 gene, are well-established [1]. This case illustrates the importance of consanguinity, a major factor in the inheritance of autosomal recessive disorders. While the patient’s brother, who has CN-II, exhibits a milder phenotype and no psychiatric manifestations, this phenotypic variability highlights the role of genetic modifiers and environmental factors in determining outcomes [6]. Importantly, emerging research indicates an increased prevalence of UGT1A1 mutations in psychiatric populations, particularly in those with schizophrenia [7]. This overlap suggests that bilirubin dysregulation may share genetic pathways with neuropsychiatric disorders, warranting further investigation into gene-environment interactions and their impact on neurodevelopment.

Impact of liver transplantation on psychiatric outcomes

Liver transplantation, the mainstay treatment for CN-I, normalizes bilirubin levels and mitigates the risk of kernicterus [3]. However, the procedure itself introduces new challenges, particularly psychiatric complications. Post-transplant patients are at increased risk for conditions such as depression, anxiety, and psychosis, driven by factors including immunosuppressive therapy, metabolic changes, and psychosocial stress [8]. In this case, the onset of psychotic symptoms shortly after transplantation suggests a multifactorial etiology. Pre-existing bilirubin-induced neurotoxicity, coupled with the stress of transplantation, may have exacerbated vulnerabilities to schizophrenia. This highlights the need for proactive psychiatric monitoring and support in transplant recipients, particularly those with a history of metabolic or neurological disorders.

Insights from animal models

Animal studies provide critical evidence supporting the connection between bilirubin metabolism and neuropsychiatric disorders. Gunn rats, which lack functional UGT1A1 enzymes, exhibit behavioral phenotypes akin to schizophrenia, including cognitive impairments and altered sensorimotor gating [1]. These findings reinforce the hypothesis that hyperbilirubinemia contributes to psychiatric symptoms through oxidative stress and neuroinflammation. Importantly, these models suggest potential therapeutic interventions, such as antioxidant treatments or drugs targeting neuroinflammatory pathways. Translational research leveraging these insights could guide the development of novel therapies for patients with metabolic-psychiatric comorbidities.

Neuroimaging and biomarker development

Recent advancements in neuroimaging offer promising avenues for understanding the long-term effects of hyperbilirubinemia on brain structure and function. Techniques such as diffusion tensor imaging and magnetic resonance spectroscopy can reveal subtle changes in white matter integrity and metabolic activity, which are likely impacted by bilirubin neurotoxicity [4]. Additionally, the observation that bilirubin levels fluctuate during acute psychotic episodes raises the possibility of using bilirubin as a biomarker to monitor disease progression and treatment response [2]. This dual role as a biomarker and therapeutic target positions bilirubin as a focal point in the integrated management of metabolic and psychiatric disorders.

Integrated management and public health implications

The complex interplay between metabolic and psychiatric disorders, as exemplified by this case, underscores the need for multidisciplinary care. Early screening for hyperbilirubinemia in at-risk populations, particularly in consanguineous families, could facilitate early diagnosis and intervention, potentially mitigating long-term complications. Genetic counseling for families affected by UGT1A1 mutations is another critical component of public health strategies. Clinically, integrated management approaches that combine liver-directed therapies with targeted psychiatric care - such as antioxidants, neuroprotective agents, and personalized psychopharmacology - hold significant promise for improving outcomes [5].

Implications for future research

The findings from this case open several avenues for future research. Longitudinal studies tracking bilirubin levels and psychiatric symptoms in individuals with CN-I and related disorders could clarify the causal relationships and identify early markers of vulnerability. Investigating the genetic overlap between bilirubin metabolism and neuropsychiatric disorders may yield insights into shared pathways, guiding the development of precision medicine approaches. Furthermore, translational research using animal models, combined with advanced neuroimaging, could inform the design of targeted interventions to mitigate the neurotoxic effects of hyperbilirubinemia. Such investigations would help clarify whether bilirubin dysregulation serves merely as a marker of these conditions or plays a more active role in their development.

## Conclusions

The presented case emphasizes the need for a deeper understanding of the intersection between metabolic disorders and psychiatric conditions. The co-occurrence of Crigler-Najjar syndrome type I and schizophrenia in this patient suggests that bilirubin dysregulation could be an important factor in the pathogenesis of psychiatric disorders. Given the documented neurotoxic effects of bilirubin and its role in oxidative stress, further studies should investigate whether early intervention in hyperbilirubinemia could reduce the risk or severity of psychiatric manifestations. From a prevention and early-intervention perspective, this case also underlines the potential role of premarital counselling clinics and genetic services, particularly in consanguineous populations, in identifying at-risk couples through targeted molecular testing and broader genomic or karyotypic evaluation, thereby allowing prediction, anticipation, and timely management of severe metabolic disorders such as CN-I. Additionally, exploring the genetic links between metabolic disorders and neuropsychiatric conditions could provide valuable insights, potentially leading to more integrated approaches in diagnosis and treatment.
